# Effects of Chronic Exposure to Microcystin-LR on Kidney in Mice

**DOI:** 10.3390/ijerph16245030

**Published:** 2019-12-10

**Authors:** Xiping Yi, Shuaishuai Xu, Feiyu Huang, Cong Wen, Shuilin Zheng, Hai Feng, Jian Guo, Jihua Chen, Xiangling Feng, Fei Yang

**Affiliations:** 1 Department of Occupational and Environmental Health, Xiangya School of Public Health, Central South University, 110 Xiangya Road, Changsha 410078, China; yixp176911007@csu.edu.cn (X.Y.); xsszll@csu.edu.cn (S.X.);; 2 Key Laboratory of Environmental Medicine Engineering, Ministry of Education, School of Public Health Southeast University, Nanjing 210009, China; 3 Key Laboratory of Hunan Province for Water Environment and Agriculture Product Safety, Central South University, Changsha 410083, China

**Keywords:** microcystin-LR, chronic oral exposure, nephrotoxicity, mice

## Abstract

Microcystin-LR (MC-LR) is a potent hepatotoxin, but a few studies suggested that it might also induce nephrotoxicity. However, nephrotoxicity induced by prolonged oral exposure to MC-LR is unknown. The aim of this study was to evaluate the potential influence of MC-LR on the kidney in mice following chronic exposure to MC-LR. In this study, we evaluated the nephrotoxicity of MC-LR in mice drinking water at different concentrations (1, 30, 60, 90, and 120 μg/L) for 6 months for the first time. The results showed that the kidney weights and the kidney indexes of mice were not altered in the MC-LR treated mice, compared with the control group. In addition, the renal function indicators revealed that the serum creatinine (SCr) levels were not significant changes after exposure to MC-LR. The blood urea nitrogen (BUN) levels were markedly decreased after exposure to 90 and 120 μg/L MC-LR for 3 months. The BUN levels were lower than that of the control group after exposure to 120 μg/L MC-LR for 6 months. The histopathological investigation revealed enlarged renal corpuscles, widened of kidney tubules, and lymphocyte infiltration in the interstitial tissue and the renal pelvis after exposure to 60, 90, and 120 μg/L MC-LR. Consequently, our results suggested that long-term exposure to MC-LR might be one important risk of kidney injury, which will provide important clues for the prevention of renal impairment.

## 1. Introduction

Cyanobacterial blooms are an increasing problem worldwide and increasingly attract public attention, since microcystins (MCs) produced by them can cause a series of water quality problems and pose hazards to human and animal health [1,2,3,4,5,6,7]. MCs are a family of cyclic heptapeptide endotoxins. Presently, approximately 200 different structural analogues of MCs have been found, and microcystin-LR (MC-LR) is reported to be the most toxic and it is the most common studied variant [8,9].

MCs are generally characterized as hepatotoxin. Liver is the primary target organ for MCs accumulation. Some studies revealed that MCs could be transported into liver cells by organic anion transporting polypeptides (OATPs) [10,11]. MC-LR-induced hepatotoxicity occurs by inhibiting protein phosphatase 1 and 2A (PP1 and PP2A) and inducing the production of ROS, followed by the destroying the cell cytoskeleton, eventually leads to liver cells necrosis and apoptosis [10,12]. In addition to the damage to the liver, some studies revealed that MCs also can cause harm to the intestines, brain, gonad, lung, and heart [13,14,15,16,17]. Recently, MCs have been reported to have penetrated renal cells in an OATPs dependent manner, subsequently promoting the accumulation of MC-LR [11,18]. Therefore, exposure to MCs may induce toxicity in the kidney [19].

Several studies revealed that MCs may produce nephrotoxicity and generate cancer-promoting effect. In cells, exposure to MC-LR could induce apoptosis in both human embryonic kidney (HEK-293) and human kidney adenocarcinoma (ACHN) cell lines by reducing G2/M phase population [20]. In aquatic organisms, renal lesions were observed. Kotak et al. [21] found that renal ultrastructural lesions, tubular epithelial necrosis, and dilation of Bowman’s space of glomeruli after injection of MC-LR into rainbow trout. Similarly, in the acute toxicity tests conducted in tilapia fish, tenca, crucian carp, and mice renal lesions produced by MC-LR were also observed [22,23,24,25]. In addition, in rats, the urinary flow and glomerular filtration rate underwent a change and the antioxidant enzymes decreased after being injected with MC-LR [26,27]. Moreover, Milutinovic et al. [28] also observed renal pathological changes after injection of MC-LR. Though exposure to MC-LR could result in renal injury of mammal or fish, these studies provide mostly data on effects after intraperitoneal injection of MCs which is an environmentally irrelevant route of exposure.

Therefore, to better assess human health risk to the environmental toxin, we firstly established a long-term oral exposure mode for MC-LR and evaluated the potential influence of chronic exposure to MC-LR on the renal biological function and pathological structure in mice. The results of this study would be conducive to assessing the health risk caused by MC-LR.

## 2. Materials and Methods

### 2.1. Chemicals

MC-LR (purity ≥ 95%) was purchased from Taiwan Algal Science Inc. (Taiwan) and stored at −20 °C. Dimethyl sulfoxide (DMSO) (purity of ≥99.7%) was obtained from Sigma-Aldrich (St. Louis, MO, USA). All other chemicals were of analytical grade and were provided by common commercial suppliers.

### 2.2. Animals and Experimental Design

The experiments were performed on normal male C57BL/6 mice (6–8 weeks old and 20–22 g in size), provided by the Experimental Animal Center of Central South University. All of the mice were acclimated to the environmental conditions for 14 days before the toxicological experiment was performed. The animals were housed in a temperature- and humidity-controlled room (24 ± 3 °C temperature and 55 ± 5% humidity) with a 12 h light and dark cycle. A total of 120 animals were randomly divided into the following six groups (n = 20): five treatment groups (exposure to MC-LR at 1, 30, 60, 90, and 120 μg/L in 0.012% (v/v) DMSO for drinking water, respectively) and one solvent (0.012% DMSO) control group. Water and rodent chow were freely available to mice. The animals in each of the groups were subjected to MC-LR exposure for 3 and 6 months, respectively; 10 animals were used in each of the examined exposure times. Five mice were randomly selected to detect their renal function indicators. Body weights were measured once every two weeks during the study. The animals were sacrificed after taken blood from eyeball at the end of the MC-LR exposure treatment, including their matched control. All of the procedures animal rights were approved by the Animal Care and Use Committee of the Central South University (identification code: XYGW-2018-41; date of approval: 10 November 2018).

### 2.3. Determination of Renal Function Indicators

The blood samples were collected from eyeball of the mice, and the serum was separated following centrifugation at 4000× *g* for 30 min. One part of the serum was kept at low temperature immediately and delivered to laboratory for detecting the concentration of the serum creatinine (SCr) and blood urea nitrogen (BUN), which are markers for nephrotoxic, adopting the biochemical automatic detector (HITACHI 7600-020 Japan). 

### 2.4. Histological Observations

Kidney tissues were immediately isolated and weighed after animals were killed. The blood stains were washed out with phosphate-buffered solution (PBS), and part of the kidney tissue from each animal was fixed with 4% paraformaldehyde (PFA), then trimmed and embedded in paraffin. Four-μm-thick sections were stained with hematoxylin and eosin (H&E). The residual kidney tissue was kept at −80 °C. The sections were observed and photographed with the Invert microscope (Motic, AE31).

### 2.5. Statistical Analysis

Statistical analyses were performed using IBM SPSS Statistics 23.0 (IBM SPSS Inc., Chicago, IL, USA). All of values were expressed as the mean ± standard deviation (SD). One-way analysis of variance (ANOVA) was applied to determine differences between groups, followed by Dunnett’s *t*-test. Curvilinear regression and Spearman correlation analysis were further used to evaluate relationships between the MC-LR concentration and the kidney weight, the kidney index, the BUN and SCr levels. Meanwhile, the multiple linear regression models was used to determine the regression coefficients. Results of all hypothesis tests with *p*-values < 0.05 (two-sided) were considered statistically significant.

## 3. Results

### 3.1. Body Weight and Kidney Index

**Figure 1:**
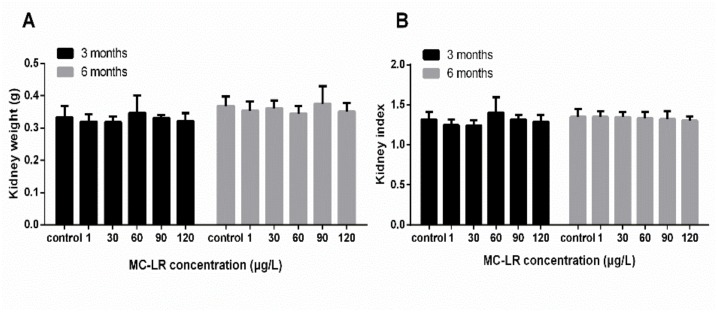
Toxic effects of MC-LR on kidney in mice. Mice were given drinking water containing various concentrations MC-LR (1, 30, 60, 90, and 120 µg/L) for 3 and 6 persistent months. (**A**) Values of kidney weight at different groups; (**B**) Values of kidney index at different groups. kidney index = (double kidney weight/body weight) × 100. The data were expressed as the mean ± SD (n = 10).

**Figure 2:**
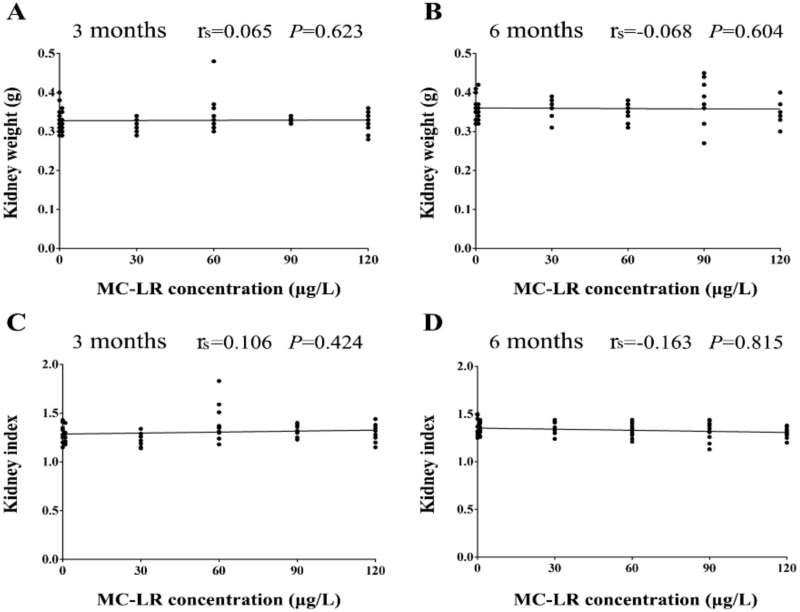
Correlation between the MC-LR concentrations and the kidney weight (**A**,**B**), the kidney index (**C**,**D**). Mice were given drinking water containing various concentrations MC-LR (1, 30, 60, 90, and 120 µg/L) for 3 and 6 months. The black dots signify the observed values of the kidney weight and the kidney index in mice (n = 10). The r_s_ signify Spearman correlation coefficients. *p* < 0.05 was considered statistically significant.

No death and symptoms in MC-LR treated mice were recorded during the 3-month or 6-month experiment. Following exposure to MC-LR for 3 and 6 months, the kidney weight of mice treated with various concentrations MC-LR (1, 30, 60, 90, and 120 μg/L) was no significant differences in comparison with the control group. The kidney indexes were also not altered after exposure to MC-LR (Figure 1). The results of Spearman correlation analysis showed that there was no a significant correlation between the MC-LR concentrations and the kidney weight, the kidney index (Figure 2). In addition, in the multiple linear regression models the levels of the kidney weight, and the kidney index were also no associated with the MC-LR concentrations after adjustment for exposure time.

### 3.2. Renal Function Indicators

**Figure 3:**
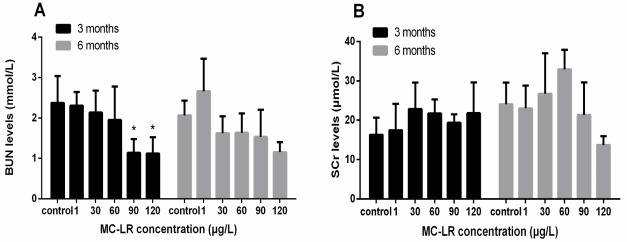
Levels of BUN and SCr after exposure to different concentrations of MC-LR. Mice were given drinking water containing various concentrations MC-LR (1, 30, 60, 90, and 120 µg/L) for 3 and 6 months. (**A**) Values of the BUN levels at different groups; (**B**) Values of the SCr levels at different groups. The values were presented as the mean ± SD (n = 5). * *p* < 0.05, vs. control.

**Figure 4:**
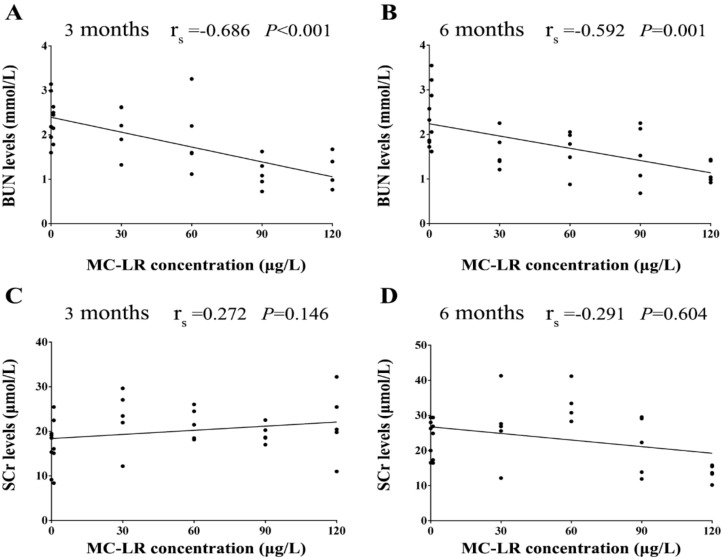
Correlation between the MC-LR concentrations and the BUN levels (**A**,**B**), the SCr levels (**C**,**D**). Mice were given drinking water containing various concentrations MC-LR (1, 30, 60, 90, and 120 µg/L) for 3 and 6 months. The black dots signify the observed values of the kidney weight and the kidney index in mice (n = 5). The r_s_ signify Spearman correlation coefficients. *p* < 0.05 was considered statistically significant.

The BUN levels were markedly decreased after exposure to 90 μg/L and 120 μg/L MC-LR for 3 months. The results of Student’s *t*-test showed that the BUN levels were lower than that of the control group after exposure to 120 μg/L MC-LR for 6 months. Following chronic exposure to MC-LR for 3 and 6 months, the SCr levels were no significant altered (Figure 3). The results of Spearman correlation analysis showed that there was a moderately negative correlation between the BUN levels and the MC-LR concentration. In addition, in the multiple linear regression models the BUN levels was associated with the MC-LR concentrations after adjustment for exposure time (β = −0.01; *p* < 0.001). There was no a significant correlation between the SCr levels and the MC-LR concentrations (Figure 4).

### 3.3. Histological Observation of Kidneys

**Figure 5:**
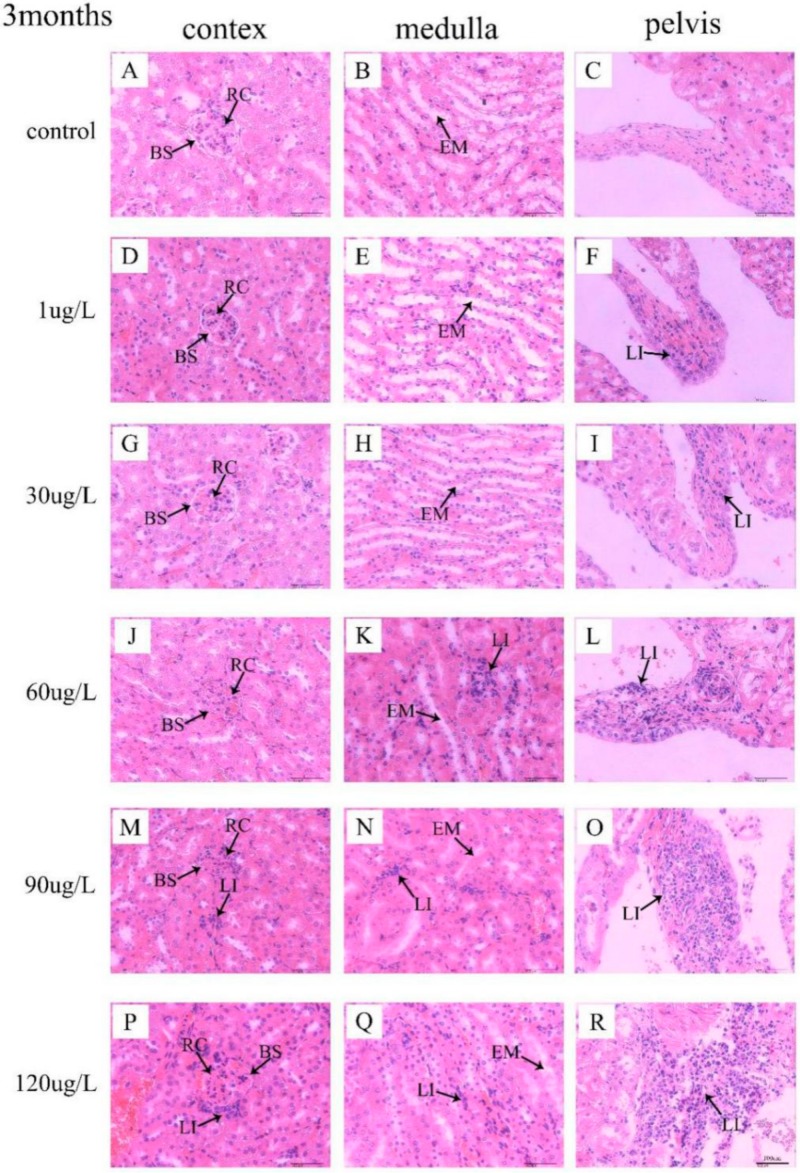
Histopathology analysis of kidney tissues from the mice treated with MC-LR for 3 months. The structure of histological sections of kidney was observed by H&E staining. Bar = 100 µm means original magnification × 400. (**A**–**C**) were control group; (**D**–**R**) were MC-LR groups. RC: renal corpuscles; BS: Bowman’s space; LI: lymphocytes infiltrate; EM: eosinophilic material.

**Figure 6:**
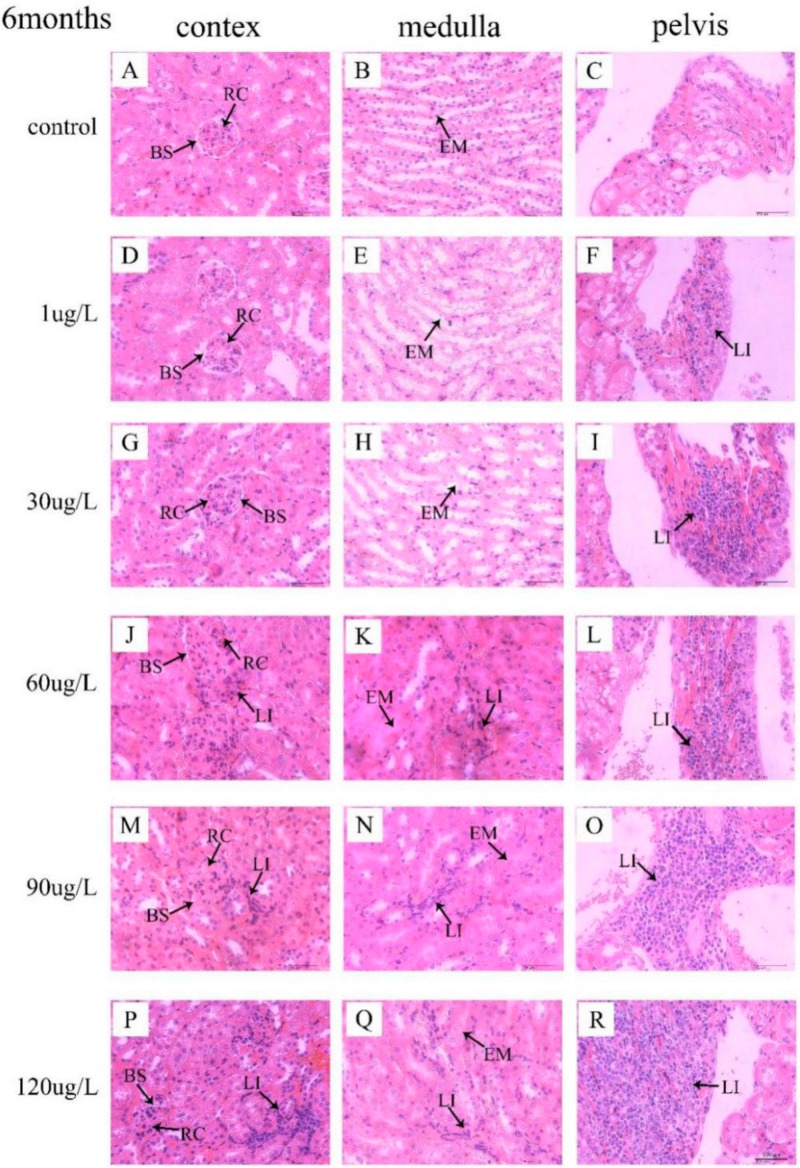
Histopathology analysis of kidney tissues from the mice treated with MC-LR for 6 months. The structure of histological sections of kidney was observed by H&E staining. Bar = 100 µm means original magnification × 400. (**A**–**C**) were control group; (**D**–**R**) were MC-LR groups. RC: renal corpuscles; BS: Bowman’s space; LI: lymphocytes infiltrate; EM: eosinophilic material.

In the control group, no significant lesions were observed in mice kidney. No remarkable structure changes of kidney in mice following exposure to 1 μg/L and 30 μg/L MC-LR were also observed. Nevertheless, following exposure to 60 μg/L MC-LR for 3 months, enlarged the renal corpuscles (RC) with compressed Bowman’s space (BS) are seen (Figure 5J). Following exposure to MC-LR for 6 months, obvious lymphocytes infiltrate (LI) in interstitial tissue were detected in mice treated with 60 μg/L MC-LR, with dilated the renal tubule (Figure 6J,K). Moreover, following exposure to 90 μg/L and 120 μg/L MC-LR, we observed that numerous renal corpuscles were enlarged with compressed Bowman’s space (BS) in the kidney cortex (Figure 5M,P and Figure 6M,P) and renal tubules was widened and filled with eosinophilic material (EM) (Figure 5N,Q and Figure 6N,Q). In addition, with the increase of exposure concentration of MC-LR in mice, lymphocyte infiltration in renal pelvis increased (Figure 5F,I,L,O,R and Figure 6F,I,L,O,R). Those mice exposed to MC-LR for 6 months displayed more lymphocytes than the 3-month groups. 

## 4. Discussion

Some previous studies have confirmed hepatotoxicity, neurotoxicity, gastrointestinal toxicity, and reproductive toxicity of MC-LR [29,30,31,32]. However, the study of nephrotoxicity induced by prolonged oral exposure to MC-LR is rare. In this study, we observed that MC-LR was associated with renal-function impairment. To our knowledge, this is the first animal experiments with prolonged oral exposure to high-dose MC-LR to provide experimental evidence of positive association of environmental exposure to MC-LR with renal-function impairment.

In mice chronic exposure models (3 and 6 months), the kidney weights and the kidney indexes of mice were not altered when they were exposed to various concentrations MC-LR (1, 30, 60, 90, and 120 μg/L), which was in accordance with previous findings that the mean of the kidney weights and the kidney indexes showed no alterations after exposure to MC-LR [33,34]. It has been reported that MC-LR was 30–100 times less toxic via oral ingestion than via intraperitoneal injection [35]. In addition, Adamovsky et al. [36] found that oral exposure of rats to high doses of microcystins in the diet induced detoxification activities by stimulating phase II enzymes GST and GR. Therefore, we may conjecture that no change of the kidney weights and the kidney indexes in mice may be due to the lower toxicity of oral exposure and the specific detoxification mechanism to adapt to the toxin exposure.

In this study, the BUN levels were markedly decreased after exposure to 90 μg/L and 120 μg/L MC-LR for 3 months. No other significant changes in the SCr levels were observed in the mice given MC-LR at any time point. Blood biochemical indicators are useful and sensitive to the diagnosis of disease and the monitoring of the physiological state of exposed to toxic substances. BUN and SCr are nitrogenous organic compounds and the end products of protein metabolism. When renal function was normal, these small molecules are filtered from the glomerulus. However, the filtering capability of the glomerulus decreases, and the content of BUN and SCr increases when the kidney suffers from lesions. This increase in serum levels could often be used as an indicator of kidney dysfunction [37]. Interestingly, in the present study, the BUN levels was decreased after exposure with 90 μg/L and 120 μg/L MC-LR for 3 months. It has been reported that the activity of AST was decreased after long-term exposure of the carp to cyanobacteria extract and the activity of ALT was decreased after exposure of rats to MCs, which may be attributed to certain tolerance and adaptation of the organism through detoxification mechanisms [36,38,39]. Therefore, we may infer that the decrease of the BUN levels may be due to the detoxification mechanisms. However, the mechanisms of the BUN levels decrease following prolonged MC-LR exposure remained to be further investigated. In addition, the BUN levels were lower than that of the control group after exposure to 120 μg/L MC-LR for 6 months. We speculate that the chronic effect can be worse with the experimental time increasing. In our study, no significant changes were observed in the SCr levels in the mice after exposure to MC-LR, which was in accordance with previous findings that the mice given MC-LR in drinking water did not show significant changes in the SCr levels [34]. It illustrates that mice might have adapted to the toxin exposure. However, this reason needs to be further studied. We speculate that renal-function impairment would occur as MC-LR exposure time increases.

Normal tissue is an important factor in the physiological function of organs. Kotak et al. [21] revealed that renal lesions consisted of coagulative tubular necrosis with a dilation of Bowman’s space after intraperitoneally administered MC-LR in rainbow trout (*Oncorhynchus mykiss*). Li et al. [23] revealed that kidney was injured by destroying renal corpuscle and renal tubules after intraperitoneal injection of extracted microcystins in omnivorous crucian carp (*Carassius auratus*). In our study, pathological lesions were observed and characterized by enlarged renal corpuscles with compressed Bowman’s space, widened of kidney tubules, and lymphocyte infiltration in the interstitial tissue and the renal pelvis which were consistent with the findings of previous studies [40]. Combined with the findings of previous studies, it can be concluded that exposure to MC-LR could negatively affect kidney through pathological lesions. In addition, epidemiological study suggested that microcystins might be one important risk of renal-function impairment [41]. Therefore, prolonged exposure to microcystins may lead to chronic kidney disease (CKD). CKD is considered as a multifactorial disease caused by genetic and environmental factors [42]. CKD has become a major public health problem among adults worldwide, whose main characterization is renal function impairment [43]. However, the possible mechanisms of MC-LR induced nephrotoxicity remained to be further investigated.

Regarding strengths and limitations, this study with prolonged oral exposure to MC-LR reported the association of MC-LR exposure with renal injury and provided experimental evidence of such toxin-induced renal injury. However, the following limitations need to be overcome in future research: first of all, we did not evaluate the internal exposure levels of MC-LR. Second, we did not do further experiments to verify the results in this article.

## 5. Conclusions

Our study demonstrated that the chronic oral exposure to MC-LR could change the BUN levels and destroy the renal structure in mice. MC-LR exposure is one important risk of kidney injury. These results provide important clues for future research and make contribution to the prevention of kidney diseases caused by MC-LR.

## References

[1] Svirčev Z., Drobac D., Tokodi N., Mijovic B., Codd G.A., Meriluoto J. (2017). Toxicology of microcystins with reference to cases of human intoxications and epidemiological investigations of exposures to cyanobacteria and cyanotoxins. Arch. Toxicol..

[2] Svirčev Z., Lujić J., Marinović Z., Drobac D., Tokodi N., Stojiljković B., Meriluoto J. (2015). Toxicopathology Induced by Microcystins and Nodularin: A Histopathological Review. J. Environ. Sci. Health C Environ. Carcinog. Ecotoxicol. Rev..

[3] Yang S., Chen L., Wen C., Zhang X., Feng X., Yang F. (2017). MicroRNA expression profiling involved in MC-LR-induced hepatotoxicity using high-throughput sequencing analysis. J. Toxicol. Environ. Health Part A.

[4] Cao L., Huang F., Massey I.Y., Wen C., Zheng S., Xu S., Yang F. (2019). Effects of Microcystin-LR on the Microstructure and Inflammation-Related Factors of Jejunum in Mice. Toxins (Basel).

[5] Massey I.Y., Yang F., Ding Z., Yang S., Guo J., Tezi C., Al-Osman M., Kamegni R.B., Zeng W. (2018). Exposure routes and health effects of microcystins on animals and humans: A mini-review. Toxicon.

[6] Cao L., Massey I.Y., Feng H., Yang F. (2019). A Review of Cardiovascular Toxicity of Microcystins. Toxins (Basel).

[7] Wei J., Xie X., Huang F., Xiang L., Wang Y., Han T., Massey I.Y., Liang G., Pu Y., Yang F. (2019). Simultaneous Microcystis algicidal and microcystin synthesis inhibition by a red pigment prodigiosin. Environ. Pollut..

[8] Meriluoto J., Spoof L., Codd G.A. (2017). Appendix 3: Tables of Microcystins and Nodularins.

[9] Du X., Liu H., Yuan L., Wang Y., Ma Y., Wang R., Chen X., Losiewicz M.D., Guo H., Zhang H. (2019). The Diversity of Cyanobacterial Toxins on Structural Characterization, Distribution and Identification: A Systematic Review. Toxins (Basel).

[10] Fujiki H., Suganuma M. (2011). Tumor promoters--microcystin-LR, nodularin and TNF-alpha and human cancer development. Anticancer Agents Med. Chem..

[11] Hagenbuch B., Meier P.J. (2003). The superfamily of organic anion transporting polypeptides. Biochim. Biophys. Acta.

[12] Clark S.P., Davis M.A., Ryan T.P., Searfoss G.H., Hooser S.B. (2016). Hepatic Gene Expression Changes in Mice Associated with Prolonged Sublethal Microcystin Exposure. Toxicol. Pathol..

[13] Hou J., Su Y., Lin W., Guo H., Xie P., Chen J., Gu Z., Li L. (2017). Microcystin-LR retards gonadal maturation through disrupting the growth hormone/insulin-like growth factors system in zebrafish. Ecotoxicol. Environ. Saf..

[14] Martins N.D., Colvara W.A., Rantin F.T., Kalinin A.L. (2011). Microcystin-LR: How it affects the cardio-respiratory responses to hypoxia in Nile tilapia, Oreochromis niloticus. Chemosphere.

[15] Wang C., Gu S., Yin X., Yuan M., Xiang Z., Li Z., Cao H., Meng X., Hu K., Han X. (2016). The toxic effects of microcystin-LR on mouse lungs and alveolar type II epithelial cells. Toxicon.

[16] Yan W., Li L., Li G., Zhao S. (2017). Microcystin-LR induces changes in the GABA neurotransmitter system of zebrafish. Aquat. Toxicol..

[17] Botha N., Venter M.V.D., Downing T.G., Shephard E.G., Gehringer M.M. (2004). The effect of intraperitoneally administered microcystin-LR on the gastrointestinal tract of Balb/c mice. Toxicon.

[18] Jia J., Luo W., Lu Y., Giesy J.P. (2014). Bioaccumulation of microcystins (MCs) in four fish species from Lake Taihu, China: Assessment of risks to humans. Sci. Total Environ..

[19] Menezes C., Elsa E.V.A. (2013). The Kidney Vero-E6 Cell Line: A Suitable Model to Study the Toxicity of Microcystins. Tech.

[20] Piyathilaka M.A., Pathmalal M.M., Tennekoon K.H., De Silva B.G., Samarakoon S.R., Chanthirika S. (2015). Microcystin-LR-induced cytotoxicity and apoptosis in human embryonic kidney and human kidney adenocarcinoma cell lines. Microbiology.

[21] Kotak B.G., Semalulu S., Fritz D.L., Prepas E.E., Hrudey S.E., Coppock R.W. (1996). Hepatic and renal pathology of intraperitoneally administered microcystin-LR in rainbow trout (Oncorhynchus mykiss). Toxicon.

[22] Atencio L., Moreno I., Jos A., Pichardo S., Moyano R., Blanco A., Camean A.M. (2008). Dose-dependent antioxidant responses and pathological changes in tenca (Tinca tinca) after acute oral exposure to Microcystis under laboratory conditions. Toxicon.

[23] Li L., Xie P., Lei H., Zhang X. (2013). Renal accumulation and effects of intraperitoneal injection of extracted microcystins in omnivorous crucian carp (*Carassius auratus*). Toxicon.

[24] Molina R., Moreno I., Pichardo S., Jos A., Moyano R., Monterde J.G., Camean A. (2005). Acid and alkaline phosphatase activities and pathological changes induced in Tilapia fish (Oreochromis sp.) exposed subchronically to microcystins from toxic cyanobacterial blooms under laboratory conditions. Toxicon.

[25] Wu J., Yang L., Zhang X., Li Y., Wang J., Zhang S., Liu H., Huang H., Wang Y., Yuan L. (2018). MC-LR induces dysregulation of iron homeostasis by inhibiting hepcidin expression: A preliminary study. Chemosphere.

[26] Moreno I., Pichardo S., Jos A., Gomez-Amores L., Mate A., Vazquez C.M., Camean A.M. (2005). Antioxidant enzyme activity and lipid peroxidation in liver and kidney of rats exposed to microcystin-LR administered intraperitoneally. Toxicon.

[27] Nobre A.C., Coelho G.R., Coutinho M.C., Silva M.M., Angelim E.V., Menezes D.B., Fonteles M.C., Monteiro H.S. (2001). The role of phospholipase A(2) and cyclooxygenase in renal toxicity induced by microcystin-LR. Toxicon.

[28] Milutinovic A., Zivin M., Zorc-Pleskovic R., Sedmak B., Suput D. (2003). Nephrotoxic effects of chronic administration of microcystins -LR and -YR. Toxicon.

[29] Florczyk M., Łakomiak A., Woźny M., Brzuzan P. (2014). Neurotoxicity of cyanobacterial toxins. Environ. Biotechnol..

[30] Lone Y., Koiri R.K., Bhide M. (2015). An overview of the toxic effect of potential human carcinogen Microcystin-LR on testis. Toxicol. Rep..

[31] Wu J.X., Huang H., Yang L., Zhang X.F., Zhang S.S., Liu H.H., Wang Y.Q., Yuan L., Cheng X.M., Zhuang D.G. (2018). Gastrointestinal toxicity induced by microcystins. World J. Clin. Cases.

[32] Woolbright B.L., Williams C.D., Ni H., Kumer S.C., Schmitt T., Kane B., Jaeschke H. (2017). Microcystin-LR induced liver injury in mice and in primary human hepatocytes is caused by oncotic necrosis. Toxicon.

[33] Heinze R. (1999). Toxicity of the cyanobacterial toxin microcystin-LR to rats after 28 days intake with the drinking water. Environ. Toxicol..

[34] Ueno Y., Makita Y., Nagata S., Tsutsumi T., Yoshida F., Tamura S., Sekijima M., Tashiro F., Harada T., Yoshida T. (1999). No chronic oral toxicity of a low dose of microcystin-LR, a cyanobacterial hepatotoxin, in female BALB/c mice. Environ. Toxicol..

[35] Fawell J.K., Mitchell R.E., Everett D.J., Hill R.E. (1999). The toxicity of cyanobacterial toxins in the mouse: I microcystin-LR. Hum. Exp. Toxicol..

[36] Adamovsky O., Palikova M., Ondrackova P., Zikova A., Kopp R., Mares J., Pikula J., Paskerova H., Kohoutek J., Blaha L. (2013). Biochemical and histopathological responses of Wistar rats to oral intake of microcystins and cyanobacterial biomass. Neuroendocrinol. Lett..

[37] Xiangyan L., Fanyun K., Haiqing Z., Renxian T., Kuiyang Z. (2011). The duplication and identification of anti-glomerular basement membrane (GBM) nephritis model in mice. Acta Acad. Med. Xuzhou.

[38] Palikova M., Navratil S., Krejci R., Sterba F., Tichy F., Kubala L., Marsalek B., Blaha L. (2004). Outcomesof repeated exposure of the carp (*Cyprinus carpio* L.) to cyanobacteria extract. ACTA Vet. Brno.

[39] Solter P., Liu Z.L., Guzman R. (2000). Decreased hepatic ALT synthesis is an outcome of subchronic microcystin-LR toxicity. Toxicol. Appl. Pharmacol..

[40] Milutinovic A., Zorc-Pleskovic R., Zivin M., Vovk A., Sersa I., Suput D. (2013). Magnetic resonance imaging for rapid screening for the nephrotoxic and hepatotoxic effects of microcystins. Mar. Drugs.

[41] Lin H., Liu W., Zeng H., Pu C., Zhang R., Qiu Z., Chen J., Wang L., Tan Y., Zheng C. (2016). Determination of Environmental Exposure to Microcystin and Aflatoxin as a Risk for Renal Function Based on 5493 Rural People in Southwest China. Environ. Sci. Technol..

[42] Estrella M.M., Li M., Tin A., Abraham A.G., Shlipak M.G., Penugonda S., Hussain S.K., Palella F.J., Wolinsky S.M., Martinson J.J. (2015). The Association Between APOL1 Risk Alleles and Longitudinal Kidney Function Differs by HIV Viral Suppression Status. Clin. Infect. Dis..

[43] Levey A.S., Atkins R., Coresh J., Cohen E.P., Collins A.J., Eckardt K.U., Nahas M.E., Jaber B.L., Jadoul M., Levin A. (2007). Chronic kidney disease as a global public health problem: Approaches and initiatives-a position statement from Kidney Disease Improving Global Outcomes. Kidney Int..

